# Biochemical Screening for Nonadherence Is Associated With Blood Pressure Reduction and Improvement in Adherence

**DOI:** 10.1161/HYPERTENSIONAHA.117.09631

**Published:** 2017-10-01

**Authors:** Pankaj Gupta, Prashanth Patel, Branislav Štrauch, Florence Y. Lai, Artur Akbarov, Gaurav S. Gulsin, Alison Beech, Věra Marešová, Peter S. Topham, Adrian Stanley, Herbert Thurston, Paul R. Smith, Robert Horne, Jiří Widimský, Bernard Keavney, Anthony Heagerty, Nilesh J. Samani, Bryan Williams, Maciej Tomaszewski

**Affiliations:** From the Department of Metabolic Medicine and Chemical Pathology, University Hospitals of Leicester NHS Trust, United Kingdom (P.G., P.P., G.S.G., P.R.S.); Department of Cardiovascular Sciences, University of Leicester, United Kingdom (P.G., P.P., F.Y.L., G.S.G., N.J.S.); National Institute for Health Research Leicester Cardiovascular Biomedical Research Unit, United Kingdom (P.G., P.P., F.Y.L., N.J.S.); Division of Cardiovascular Sciences, Faculty of Biology, Medicine, and Health, University of Manchester, United Kingdom (P.G., A.A., B.K., A.H., M.T.); 3rd Department of Medicine, Department of Endocrinology and Metabolism, Hypertension Centre (B.S., J.W.) and Institute of Forensic Medicine and Toxicology (V.M.), General University Hospital, Charles University, Prague, Czech Republic; University of Leicester, United Kingdom (A.B.); University Hospitals of Leicester NHS Trust, United Kingdom (P.S.T., A.S., H.T.); Department of Health Psychology, University College of London, United Kingdom (R.H.); Division of Medicine, Central Manchester NHS Foundation Trust, Manchester Academic Health Science Centre, United Kingdom (B.K., A.H., M.T.); Institute of Cardiovascular Science, University College London, United Kingdom (B.W.); and National Institute for Health Research, University College London Hospitals Biomedical Research Centre, United Kingdom (B.W.).

**Keywords:** adherence, antihypertensive agents, blood pressure, chromatography, liquid, hypertension

## Abstract

We hypothesized that screening for nonadherence to antihypertensive treatment using liquid chromatography-tandem mass spectrometry–based biochemical analysis of urine/serum has therapeutic applications in nonadherent hypertensive patients. A retrospective analysis of hypertensive patients attending specialist tertiary care centers was conducted in 2 European countries (United Kingdom and Czech Republic). Nonadherence to antihypertensive treatment was diagnosed using biochemical analysis of urine (United Kingdom) or serum (Czech Republic). These results were subsequently discussed with each patient, and data on follow-up clinic blood pressure (BP) measurements were collected from clinical files. Of 238 UK patients who underwent biochemical urine analysis, 73 were nonadherent to antihypertensive treatment. Their initial urinary adherence ratio (the ratio of detected to prescribed antihypertensive medications) increased from 0.33 (0–0.67) to 1 (0.67–1) between the first and the last clinic appointments. The observed increase in the urinary adherence ratio in initially nonadherent UK patients was associated with the improved BP control; by the last clinic appointment, systolic and diastolic BPs were ≈19.5 and 7.5 mm Hg lower than at baseline (*P*=0.001 and 0.009, respectively). These findings were further corroborated in 93 nonadherent hypertensive patients from Czech Republic—their average systolic and diastolic BPs dropped by ≈32.6 and 17.4 mm Hg, respectively (*P*<0.001), on appointments after the biochemical analysis. Our data show that nonadherent hypertensive patients respond to liquid chromatography–tandem mass spectrometry-based biochemical analysis with improved adherence and significant BP drop. Such repeated biochemical analyses should be considered as a therapeutic approach in nonadherent hypertensive patients.

Hypertension has become the single leading cause of global disease burden ahead of smoking and alcohol use.^1^ All cardiovascular complications of hypertension are related to poor blood pressure (BP) control.^2–4^ Despite the widespread availability of effective antihypertensive therapies, BP control is achieved only in approximately half of the Western populations.^5,6^ One of the potential explanations for this apparent paradox is the pandemic of nonadherence to antihypertensive medications.^7–10^ Recent studies show that 25% to 65% hypertensive patients do not take their BP-lowering medications as prescribed.^8,10–18^ These figures are extremely robust because they come from studies that used a direct and objective method of screening for therapeutic nonadherence.^8,10–18^ High performance liquid chromatography–tandem mass spectrometry (LC-MS/MS) provides a highly sensitive and specific detection of all commonly prescribed BP-lowering medications (or their metabolites) in urine/serum samples. Because of its simplicity, relatively low cost, and objective nature of analysis, it is a useful diagnostic test in patients with apparent lack of BP response to antihypertensive treatment.^17^

Here, we examined the potential therapeutic applications of biochemical screening for the presence of antihypertensive medications in bodily fluids. We demonstrate that nonadherent patients who undergo LC-MS/MS–based analyses exhibit an improvement in adherence and a clinically meaningful BP drop. We further show that a majority of initially nonadherent patients can successfully improve their adherence through repeated LC-MS/MS–based analysis and achieve BP targets similar to those who have been persistently adherent to treatment.

## Methods

### Data Collection and Biochemistry

#### UK Patients

We collected retrospective data from patients attending University Hospitals of Leicester NHS Trust Blood Pressure Clinic (European Society of Hypertension Centre of Excellence) between 2011 and 2014. Included in this analysis were hypertensive patients who had at least 1 biochemical screening for nonadherence to antihypertensive treatment (by LC-MS/MS of urine) during this period. Referred for the biochemical screening for nonadherence in the UK center were patients suspected to deviate from the prescribed antihypertensive therapy by their managing doctor as reported before.^19^ None of the patients asked to provide a urine sample for LC-MS/MS–based analysis refused to undertake the test. Apart from the results of LC-MS/MS–based urine analyses, we collected available basic demographic and clinical characteristics, information on prescribed antihypertensive medications, and their changes between the first (baseline) and the last clinical appointments (as defined by the timing of LC-MS/MS–based urine analysis). BP was recorded using a validated semiautomatic device (A&D Digital BP Monitor UA-767PC, A&D Instruments, Abingdon, United Kingdom). Measurements were made as per the National Institute of Health and Care Excellence Guideline 127.^20^ The 24-hour ambulatory BP monitoring was conducted using Spacelabs 90217A-1 monitors (Space Labs Healthcare, Snoqualmie, Washington) in line with the National Institute of Health and Care Excellence guidelines.^20^

On the day of their clinic appointment, patients were asked to provide a urine sample for the analysis of their adherence to antihypertensive medications.^10^ Briefly, the LC-MS/MS–based screening detects 40 of the most commonly prescribed antihypertensive medications.^10^ Samples were collected in a standard container, stored at −80°C until analysis, and examined using the Agilent Technologies 1290 High Pressure Liquid Chromatograph interfaced with an Agilent Technologies 6460 Triple Quad Mass Spectrometer (Santa Clara, CA) fitted with a jet stream electrospray source. The detection was based on the presence of the specific precursor ion to product ion transition (at least 2 for each analyte) and retention times.^10^

Patients whose baseline urine analysis by LC-MS/MS did not detect at least one of the prescribed antihypertensive medications were classified as initially nonadherent. Those whose baseline urine analysis detected all prescribed antihypertensive medications were classified as initially adherent. Patients who were initially nonadherent as defined above but whose subsequent urine analysis detected all prescribed medications were defined as converters. Patients who were initially adherent as defined above and whose subsequent urine analysis continued to show presence of all prescribed medications were classified as persistently adherent.

#### Czech Republic Patients

All hypertensive patients referred with suboptimal BP control to the Hypertension Unit at the 3rd Department of Medicine, General University Hospital, in Prague between 2010 and 2016 were included in the initial retrospective analysis of clinical notes. Included in this project were hypertensive patients diagnosed with nonadherence to antihypertensive treatment by LC-MS/MS–based analysis and at least 2 clinic appointments with recorded clinic BP values. The Czech patients were referred for LC-MS/MS–based analysis if their treating clinician found their BP control was suboptimal on the existing antihypertensive treatment.^20^ None of the patients asked to provide a blood sample for LC-MS/MS–based analysis refused. Similar to the UK cohort, information on demographic data, prescribed antihypertensives, and clinic BP values recorded at baseline and on follow-up appointments was retrieved retrospectively from the clinical files or electronic systems. The follow-up appointments were conducted either in Hypertension Unit at the 3rd Department of Medicine, General University Hospital, in Prague or in the primary care. BP measurements were taken using validated oscillometric automated devices in line with guidelines of the Czech Society of Hypertension.^21^

Serum samples were collected on the initial visit in the outpatient clinic of the Hypertension Unit. Analysis of the serum concentrations of antihypertensive medications was performed as described before.^22–25^ Serum samples were collected as a part of routine service and sent to the Toxicology laboratory of the Institute of Forensic Medicine and Toxicology. The samples were aliquoted and stored at −80°C until analysis. The LC-MS/MS was performed using Agilent Technologies 1200 Rapid Resolution Liquid Chromatography consisting of a degasser, binary pump, autosampler, and thermostatted column compartment. The mass spectrometry analysis was performed using an MDS Sciex 3200 Q142 trap triple quadrupole/linear ion trap mass spectrometer with a TurboIonSpray source.

Patients whose baseline serum analysis by LC-MS/MS did not detect at least one of the prescribed antihypertensive medications were classified as nonadherent.

The studies comply with the Declaration of Helsinki. In the UK cohort, patients were informed about the purpose of urine collection on the day of their clinical appointment. The UK patients gave a verbal consent for the biochemical screening for nonadherence, and the project was approved by University Hospitals of Leicester (audit registration number: 5944) and ratified by the local ethics committee (reference no: 17/EM/0027). Czech Republic patients provided a written consent for collection of their data in anonymized form, and the project received an institutional approval (VFN 004707/2017). The results of all biochemical analyses were sent to the appropriate clinician who informed the patients of the findings and discussed them during their clinic appointment. The form and timing of the discussion were left to the discretion of the responsible clinician.

### Statistical Analysis

Descriptive statistics are presented as counts (percentages), means (SDs), or medians (interquartile ranges). Crude comparisons of basic demographic and clinical characteristics between 2 groups (ie, initially adherent versus nonadherent patients) were conducted using Fisher exact test or *t* test as appropriate. The groups were also compared in terms of the number of prescribed and detected BP-lowering drugs, adherence ratio, and BP over follow-up appointments using mixed-effects regression models to account for the correlated nature of observations across the appointments. Continuous variables, such as BP, were log transformed, the adherence proportion data were arcsine transformed, and both analyzed using linear mixed-effects models. Variables based on counts, such as the differences in the number of prescribed and detected antihypertensive medications, were examined using Poisson mixed-effects models. Nondemographic comparisons were adjusted for age, sex, ethnicity (where appropriate), and the number of prescribed antihypertensive medications. The *R*^2^ of mixed-models was evaluated using Nakagawa and Schielzeth’s approach.^26^ All analyses were conducted using R^27^ with the use of the following R packages: lme4,^28^ data.table,^29^ and ggplot2.^30^

## Results

### General Clinical Characteristics

Of 238 UK patients included in this analysis, 165 and 73 were classified as initially adherent and nonadherent (respectively) based on the results of their first LC-MS/MS urine test. The basic demographic characteristics, as well as the percentage of attended follow-up appointments, were similar in both groups (Table 1). The clinical characteristics of 93 Czech patients whose nonadherence was confirmed by LC-MS/MS–based analysis of serum are also shown in Table 1. None of the UK or Czech patients with the initial biochemical confirmation of nonadherence to antihypertensive treatment admitted being nonadherent before the LC-MS/MS–based analysis.

**Table 1.**
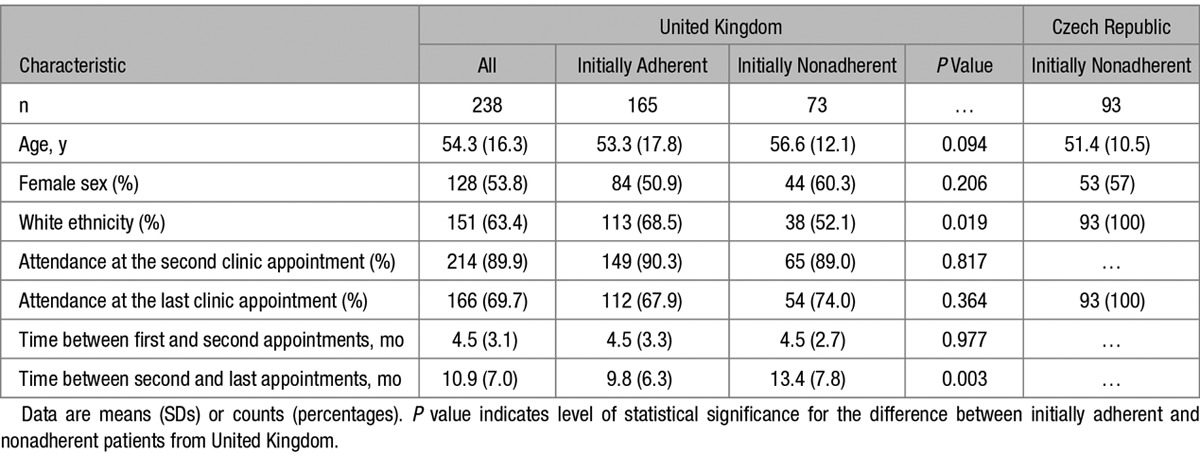
Demographic and Clinical Characteristics of Patients

### Initially Nonadherent Hypertensive Patients Show Steeper Reduction in BP on Follow-Up Appointments Than Those Who Are Initially Adherent to Antihypertensive Therapy

As expected, on the first clinic appointment, initially nonadherent patients had higher BP values than those who adhered to antihypertensive therapy (Table 2). However, there were no statistically significant differences in either clinic systolic BP (SBP) or diastolic BP (DBP) between both groups on the last clinic appointment (Table 2). This pattern of changes was replicated for both mean SBP and DBP on 24-hour ambulatory BP monitoring (Table 2). The median number of prescribed antihypertensive medications increased from 2 to 3 in the initially adherent group but remained constant at 4 in the initially nonadherent patients (Table 2). Further sensitivity analyses restricted to those with suboptimal BP control on treatment with at least 3 antihypertensive medications (including a diuretic)^31^ confirmed that the differences in BP between initially nonadherent and adherent patients followed the pattern of changes observed in the analysis of the entire cohort of hypertensives (data not shown).

**Table 2.**
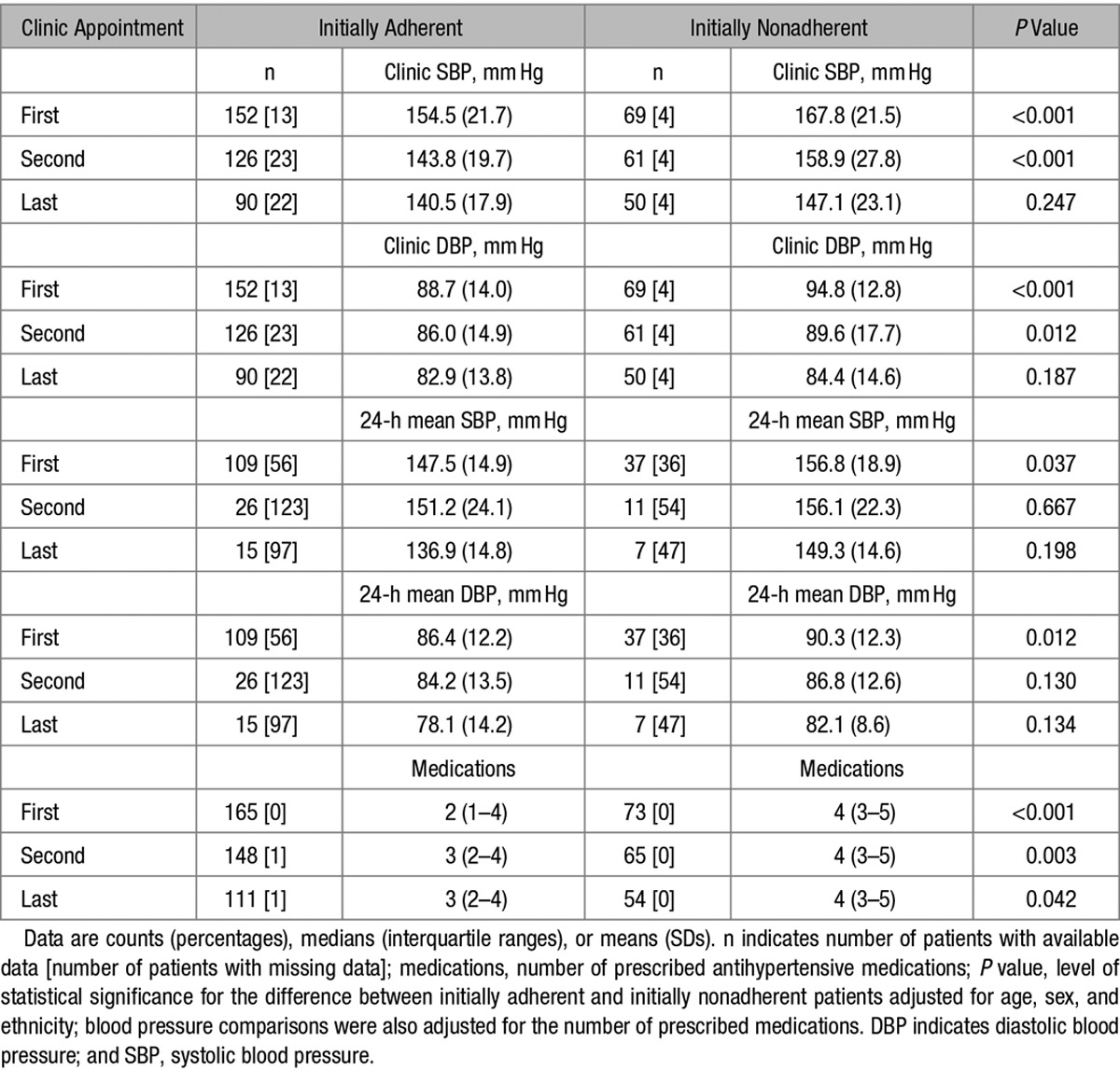
Blood Pressure Values on Follow-Up Appointments: Comparison of Initially Adherent and Initially Nonadherent Patients

### LC-MS/MS–Based Analysis Is Associated With Better Adherence to Antihypertensive Treatment and Improved BP Control in Initially Nonadherent Patients

We further examined the patterns of changes in adherence to antihypertensive therapy and BP exclusively in UK patients who were nonadherent on their first clinic appointment. Of 73 initially nonadherent patients, 30 had repeated LC-MS/MS–based urine analysis coinciding with clinic BP measurements. There were no statistically significant differences in their basic clinical characteristics when compared with the 43 nonadherent patients who did not have repeated urine analysis (data not shown). The median adherence ratio increased between their first and last appointments (Table 3). Although the median number of prescribed medications remained constant (*P*=0.906), the median number of drugs detected in urine by LC-MS/MS increased from 1 to 3 (*P*<0.001; Table 3). By the last appointment, 80% of the 30 initially nonadherent patients with follow-up LC-MS/MS–based analysis improved their urinary adherence ratio and 53.3% became fully adherent (converters). We also recorded 19.5- and 7.5-mm Hg reduction in SBP and DBP (respectively) between the patients’ first and last appointments (Table 3).

**Table 3.**
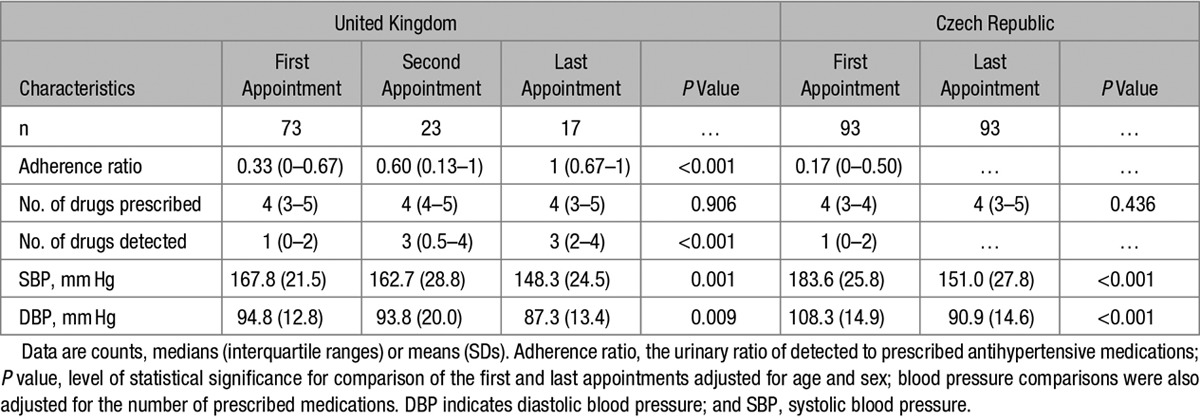
Changes in Clinic Blood Pressure and Adherence to Antihypertensive Treatment in Initially Nonadherent Patients

An additional analysis conducted in an independent sample of 93 nonadherent hypertensive patients from Czech Republic revealed even more significant reduction in BP—average SBP and DBP dropped by 32.6 and 17.4 mm Hg, respectively (*P*<0.001) on appointments after the initial LC-MS/MS–based serum analysis. This BP reduction occurred without statistically significant changes in the average number of prescribed antihypertensive medications (Table 3).

### Initially Nonadherent Patients Who Become Converters Reach BP Similar to That of Persistently Adherent Patients

Nineteen initially adherent patients had repeated LC-MS/MS–based urine analysis and remained adherent to treatment by the final clinic appointment (persistently adherent patients). Their clinical characteristics are shown in Table 4. Although the baseline clinic BP was numerically lower in persistently adherent patients than in converters, by the last appointment, both clinic SBP and DBP became statistically comparable between both groups of patients (Table 4).

**Table 4.**
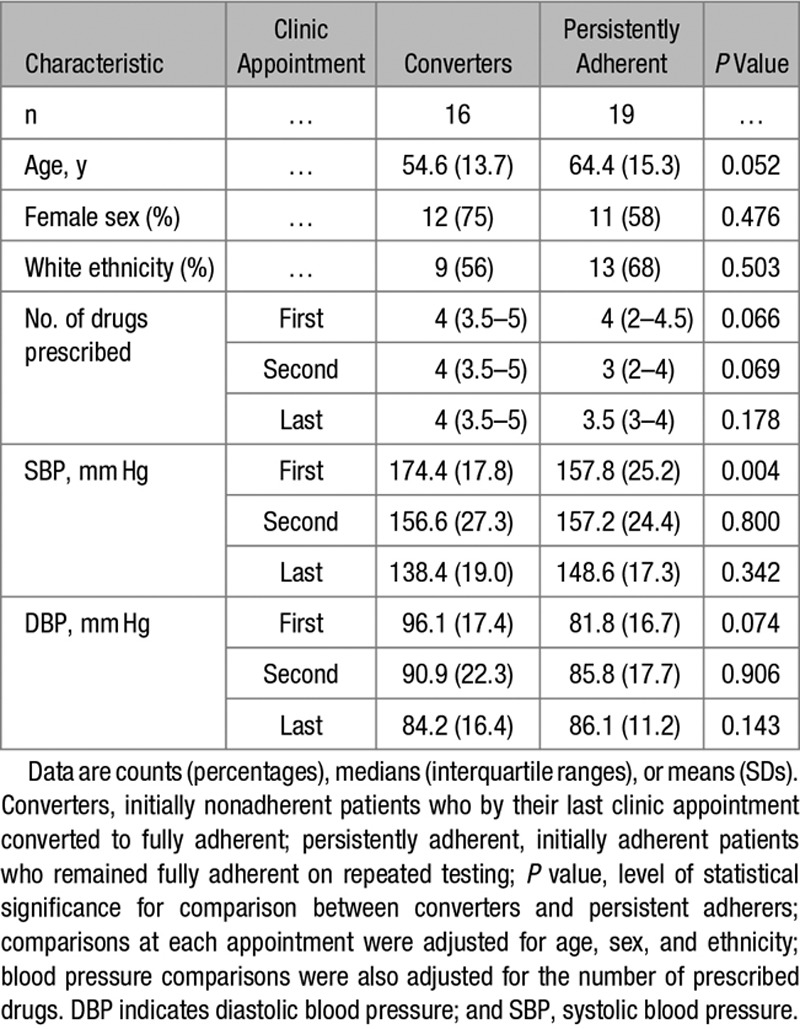
Clinical Characteristics, Adherence to Antihypertensive Treatment, and Clinic Blood Pressure in Converters and Persistently Adherent Patients

### Numeric Change in the Adherence Ratio Shows a Correlation With the Drop in BP in Initially Nonadherent Patients Who Underwent Repeated Urine Analysis and BP Measurements

We detected a significant association between the increase in the adherence ratio (on LC-MS/MS–based urine analysis) and drop in BP (on clinic measurements) in 29 UK patients who underwent repeated screening for nonadherence and had BP measurements taken on the same visits (*R*^2^=0.25, *P*=0.002 for SBP and *R*^2^=0.21, *P*=0.005 for DBP). An average increase in urinary adherence ratio of 0.5 correlated with an ≈16-mm Hg drop in SBP and 9-mm Hg drop in DBP (Figure). After adjustment for age, sex, ethnicity, and the number of prescribed drugs, the association between the increase in the urinary adherence ratio and drop in BP remained significant (*P*=0.010 for SBP and *P*=0.006 for DBP)

**Figure.**
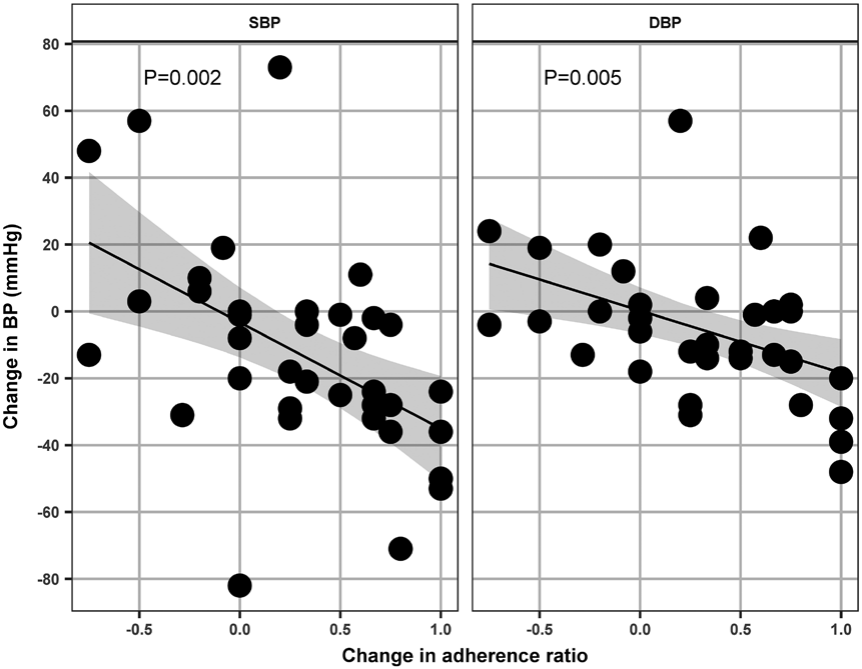
Association between changes in blood pressure and urinary adherence ratio in initially nonadherent patients. *X* axis represents the change in urinary adherence ratio (the ratio of detected to prescribed antihypertensive medications), *y* axis represents the change in clinic blood pressure (BP; mm Hg), data points are changes in BP and urinary adherence ratio between the appointments with complete information (both BP and adherence) available, *P* value indicates level of statistical significance for association between the change in urinary adherence ratio and change in systolic BP (SBP) and diastolic BP (DBP).

## Discussion

Our study provides several important insights into the potential therapeutic use of biochemical screening for nonadherence to antihypertensive treatment. First, we demonstrate that nonadherent hypertensive patients who underwent LC-MS/MS–based analysis of urine/serum and were informed of the results exhibit a significant BP drop on subsequent clinical appointments. Second, we show that repeated LC-MS/MS–based analyses have the potential to normalize adherence to antihypertensive treatment in a majority of initially nonadherent patients. Third, our data suggest that the observed improvement in BP on follow-up appointments can be explained by an improvement in urinary adherence ratio. Finally, we reveal that those who fully convert from initial nonadherence to full adherence may achieve BP levels similar to those who have been persistently adherent to treatment.

We and others previously demonstrated the diagnostic use of LC-MS/MS–based screening in detecting nonadherence to antihypertensive treatment.^8,10–12,14–18^ It also emerges that biochemical screening for nonadherence to BP-lowering therapy may be helpful in treatment of hypertension. Indeed, 16 hypertensive patients with biochemically confirmed nonadherence showed 46- and 14-mm Hg drop in clinic SBP and DBP, respectively, on follow-up in a pilot US study.^13^ We extend these observations to larger samples of patients recruited in 2 European countries. Most importantly, our study demonstrates that a reduction in BP can be explained (at least in part) through improved therapeutic adherence (as measured by the average increase in detected BP-lowering medications and the absolute increase in urinary adherence ratio).

We also show for the first time that as the directly measured adherence ratio improves over the follow-up appointments, the number of initially nonadherent patients drops, and adherence normalizes in 53.3% of initially nonadherent individuals. This means that a majority of initially nonadherent patients may convert into adherence on repeated LC-MS/MS–based analysis. The mechanisms responsible for BP reduction after LC-MS/MS–based analyses remain unclear. We anticipate that discussing the results of LC-MS/MS–based analysis break down key barriers to adherence on both the clinician’s and the patient’s side.^32,33^ In our study, the form and content of this discussion were left at the discretion of the managing physician. At University Hospitals of Leicester BP clinic, this structured discussion aims to identify and eliminate the lead reason(s) for nonadherence to antihypertensive treatment. The conceptual principles of this intervention are rooted in the apparent BP-lowering efficacy of therapeutic drug monitoring^13^ and follow the strategy proposed by National Institute of Clinical Excellence (National Institute for Health and Care Excellence) guidelines on adherence to medicines.^34^ The discussion focuses primarily on (1) polypharmacy, (2) practical difficulties (ie, forgetting, managing treatment costs, etc.) that through limitations in capability and resources affect ability to adhere, and (3) perceptions affecting the motivation to adhere (eg, beliefs that daily treatment is not necessary and concerns about harm). Such structured discussions are intended to help clinicians to explain the results of the urine analysis with the patients and to tailor adherence support through addressing the specific perceptual and practical factors influencing the patients’ motivation and ability to adhere as recommended in the National Institute for Health and Care Excellence Guidelines.^34^

The potential benefits of LC-MS/MS–based analysis in managing nonadherent hypertensive patients should not be underestimated. First, a reduction of ≈20 mm Hg in SBP achieved by repeated biochemical analysis may potentially translate into a 45% reduction in risk of coronary heart disease and ≈65% reduction in risk of stroke.^4,35^ Conversion of the majority of nonadherent hypertensive patients to adherence with a significant drop in BP would be an important breakthrough in the field, given that previous studies showed limited benefits from complex and costly interventions.^36–38^ Based on the previous simulations, 54% improvement in adherence (conversion from nonadherence to adherence) at the population level may reduce the number of strokes, myocardial infarction, kidney disease, and heart failure by ≈4.6 million with a cost reduction of ≈$39 billion.^39^ In economic terms, nonadherence to antihypertensive medications accounts for ≈$18.5 billion excess costs to the US health economy.^40^ Thus, the change of more than half of nonadherent patients to adherence patients with improvement of their BP control by ≈20 mm Hg is likely to have an important impact on health economy if the tests were used routinely and on a widespread basis. It is worth reflecting that the cost of the assay is less than a monthly supply of antihypertensive medications for some patients. Further larger, prospective randomized controlled trials are necessary to precisely quantify the efficacy of LC-MS/MS–based intervention and elucidate the factors behind the successful conversion from nonadherence to adherence to antihypertensive treatment.

We appreciate some limitations of our study. First, the patients who underwent this analysis represent a specific group of hypertensive patients with suboptimal BP control. Second, our results are based on retrospective analyses of clinical notes, and we recognize the limitation of incomplete data availability and unmeasured confounding inherent to this type of analysis. Third, information on the reasons for nonadherence was not formally a part of our data collection. However, forgetfulness emerges as one of the most common drivers of suboptimal adherence in interviews with patients conducted at University Hospitals of Leicester BP clinic. This form of nonintentional nonadherence to antihypertensive therapy was also demonstrated as one of the main barriers to adherence in other studies.^41,42^ Our study is not immune from the so-called toothbrush effect^43^—patients may have taken their medications just before their clinic appointment and thus influence their clinic BP measurement. To this end, the availability of 24-hour ambulatory BP monitoring data showing the same direction of BP changes is reassuring because this investigation was conducted at a different time point from the clinic appointment. Finally, our data provide insights into repeated snapshots of adherence rather than continuous therapeutic persistence.

## Perspectives

We show that repeated biochemical screening for nonadherence by LC-MS/MS is associated with BP reduction. We further demonstrate that a majority of nonadherent patients can be converted to full adherence by repeated testing with optimization of their BP control. To this end, we suggest that repeated LC-MS/MS–based analysis should be considered as a potential therapeutic approach to nonadherence-driven pseudoresistant hypertension. Future well-designed studies are needed to confirm these findings in prospective clinical trials and address the impact of this test on cardiovascular outcomes and its impact on the global health economy.

## Sources of Funding

B. Keavney is supported by a British Heart Foundation Personal Chair. M. Tomaszewski’s work on adherence is supported by British Heart Foundation Clinical Study (CS/17/3/32799).

## Disclosures

B. Williams has received modest honoraria for lectures from Novartis, Servier, Daiichi Sankyo, Boehringer Ingelheim, and Pfizer outside the submitted work. M. Tomaszewski received modest honoraria for lectures/presentations from Boehringer Ingelheim. The other authors report no conflicts.

## Novelty and Significance

### What Is New?

- Hypertensive patients with biochemically confirmed nonadherence to antihypertensive treatment respond to the liquid chromatography-tandem mass spectrometry analysis with a significant drop in blood pressure.
- Eighty percent of initially nonadherent hypertensive patients show an improvement in their adherence in response to initial biochemical screening.

### What Is Relevant?

- Biochemical testing for nonadherence has an important therapeutic potential.

### Summary

Biochemical screening for nonadherence to antihypertensive treatment leads to a significant improvement in blood pressure as a result of improved adherence.
